# Shrinkage-based Random Local Clocks with Scalable Inference

**DOI:** 10.1093/molbev/msad242

**Published:** 2023-11-10

**Authors:** Alexander A Fisher, Xiang Ji, Akihiko Nishimura, Guy Baele, Philippe Lemey, Marc A Suchard

**Affiliations:** Department of Statistical Science, Duke University, Durham, NC, USA; Department of Mathematics, School of Science & Engineering, Tulane University, New Orleans, LA, USA; Department of Biostatistics, Bloomberg School of Public Health, Johns Hopkins University, Baltimore, MD, USA; Department of Microbiology, Immunology and Transplantation, Rega Institute, KU Leuven, Leuven, Belgium; Department of Microbiology, Immunology and Transplantation, Rega Institute, KU Leuven, Leuven, Belgium; Department of Computational Medicine, University of California, Los Angeles, CA, USA; Department of Human Genetics, David Geffen School of Medicine, University of California, Los Angeles, CA, USA; Department of Biostatistics, Jonathan and Karin Fielding School of Public Health, University of California, Los Angeles, CA, USA

**Keywords:** Bayesian phylogenetics, shrinkage clock, random local clock, divergence time estimation, Hamiltonian Monte Carlo

## Abstract

Molecular clock models undergird modern methods of divergence-time estimation. Local clock models propose that the rate of molecular evolution is constant within phylogenetic subtrees. Current local clock inference procedures exhibit one or more weaknesses, namely they achieve limited scalability to trees with large numbers of taxa, impose model misspecification, or require a priori knowledge of the existence and location of clocks. To overcome these challenges, we present an autocorrelated, Bayesian model of heritable clock rate evolution that leverages heavy-tailed priors with mean zero to shrink increments of change between branch-specific clocks. We further develop an efficient Hamiltonian Monte Carlo sampler that exploits closed form gradient computations to scale our model to large trees. Inference under our shrinkage clock exhibits a speed-up compared to the popular random local clock when estimating branch-specific clock rates on a variety of simulated datasets. This speed-up increases with the size of the problem. We further show our shrinkage clock recovers known local clocks within a rodent and mammalian phylogeny. Finally, in a problem that once appeared computationally impractical, we investigate the heritable clock structure of various surface glycoproteins of influenza A virus in the absence of prior knowledge about clock placement. We implement our shrinkage clock and make it publicly available in the BEAST software package.

## Introduction

Molecular clock models are ubiquitous phylogenetic instruments for divergence-time estimation with applications ranging from timing placental mammal radiation (Springer et al. 2003) to estimating influenza diversity (Davidson et al. 2014). To capture clock rate variation along the lineages of a phylogeny, Thorne et al. (1998) and Rannala and Yang (2007) propose an autocorrelated, or “heritable” rate model, while others (Yoder and Yang 2000; Drummond and Suchard 2010) assume there exist, at most, a small number of “local” clocks on any given tree. Separately, Sanderson (2002) induces clock rate smoothing via a penalized likelihood approach. In each case, closely related lineages maintain similar or even identical evolutionary rates. Autocorrelated rate models are computationally appealing due to the induced smooth transition in rate from parent to child node along the tree but may inappropriately shrink large rate changes between adjacent nodes (Smith et al. 2010). On the other hand, local clock models allow large rate changes to exist but can be computationally unpalatable on large problems due to the combinatorial complexity of choosing (or learning) the number and location of local clocks. When these quantities are simultaneously learned with the tree, Drummond and Suchard (2010) call this the random local clock (RLC) model.

Due to these complications, some authors employ uncorrelated relaxed clocks such as the uncorrelated log-normal relaxed molecular clock (Drummond et al. 2006), but this generates excessive rate heterogeneity in cases where clock rate changes are thought to be more punctuated, for example between HIV subtypes (Bletsa et al. 2019). Indeed, in a variety of settings, autocorrelation between clock rates appears crucial for accurate divergence-time estimation (Lepage et al. 2007). For an in-depth review of various molecular clock models, see Ho and Duchêne (2014). Here, we propose an autocorrelated clock model where we place a Bayesian bridge shrinkage prior on the increment between parent and child log branch rates. Among various shrinkage priors in the literature, the Bayesian bridge has a unique advantage in having both a collapsed spike-and-slab representation as well as a Gaussian scale-mixture form. The first representation intuitively places large mass near zero reflecting our a priori belief that most increments should be zero but has heavy tails that allow for estimating large rate changes in an approximately unbiased manner. Like many other shrinkage priors, the Bayesian bridge includes a “global scale” nuisance parameter. In the absence of prior information, learning about this nuisance parameter typically limits the speed of inference. Polson et al. (2014) develop a framework to facilitate efficient Gibbs sampling of this nuisance parameter in a regression context and we utilize their approach here. On the other hand, the second representation of the Bayesian bridge as Gaussian scale-mixture is differentiable almost everywhere. We exploit this feature and develop an efficient Hamiltonian Monte Carlo (HMC) sampler over the space of increments that employs recent work on closed form gradient representations (Ji et al. 2020) to make our shrinkage-clock inference scalable to large trees. Crucial to our inference, we define recursive algorithms to compute the requisite joint gradient of the log-posterior in our transformed increment space with computational complexity that scales only linearly with the number of tips in the tree. We implement our method in BEAST (Suchard et al. 2018), a popular software package for reconstructing rooted, time-measured phylogenies. Due to our efficient inference machinery, our shrinkage clock achieves the tractable benefits of the autocorrelated rate model and simultaneously maintains the flexibility of more punctuated local clock models.

We examine our model under examples where identifying local clock structure is vital to accurate divergence-time estimation. For this reason, we compare our method to the state-of-the-art RLC. We demonstrate the inference speed gains of our approach versus the RLC across 90 different simulated data sets comprised of 40, 80, and 160 taxa trees. We additionally compare the accuracy of our shrinkage clock to the RLC by studying the adaptive radiation of rodents and other mammals, and demonstrate utility of the heavy-tailed Bayesian bridge shrinkage prior by comparing it to the more ubiquitous Laplace prior. Finally, we deploy our shrinkage clock to estimate the existence, location, and magnitude of host-specific clock rates in surface glycoproteins of the influenza A virus.

## Materials and Methods

### Shrinkage-based RLCs

#### Setup

Consider a rooted, bifurcating tree F with *N* tips and N−1 internal (ancestral) nodes. We index tips i=1,…,N and internal nodes i=N+1,…,2N−2. We designate node 2N−1 to be the root of the tree. Let pa(i) denote the parent of the *i*th node and let branch length $t_{i}$ connect node *i* with its parent.

#### The Relaxed Clock

Aligned molecular sequence data S evolve according to a continuous time Markov process defined by infinitesimal rate matrix Q. In our examples, Q is a 4×4 matrix that describes the relative substitution process between nucleotides along the branches in F, but in general, Q may be of larger dimension to accommodate alignments at the codon or amino acid resolution, see Yang (2014) for reference. Each site *l* of S evolves independently and identically according to Q but may have its own site-specific rate of evolution $s_{l}$. A priori we specify that $E[s_{l}]=1$. Under the relaxed clock model, the transition probability matrix for branch *i*,


(1) $$P_{i}=\exp\;\{\rho_{i}t_{i}s_{l}Q\},$$

where branch-rate multiplier $\rho_{i}$ is the number of expected substitutions per unit time. To resolve identifiability issues between the height of the tree and branch-rate multipliers $\rho =\{\rho_{1},\ldots ,\rho_{2N-2}\}$, we employ the rescaling proposed by Drummond and Suchard (2010). Under this transform, each branch-rate multiplier is the product of branch-specific clock rate $r_{i}$ and global substitution rate *γ* scaled by the inverse of total expected substitutions per total tree time,


(2) $$\rho_{i}=\gamma r_{i}\frac{\sum_{k}t_{k}}{\sum_{k}r_{k}t_{k}}.$$

This results in one fewer degree of freedom since


(3) $$\frac{\sum_{i}\rho_{i}t_{i}}{\sum_{i}t_{i}}=\gamma .$$

For heterochronous data, we estimate *γ*, but for ultrametric studies where the height of the tree is not identifiable we fix γ=1.

#### Autocorrelated Shrinkage Clock

We assume clock rates $r=\{r_{1},\ldots ,r_{2N-2}\}$ are autocorrelated and model the incremental difference $\phi_{i}$ between branch *i*’s clock rate and its parent lineage clock rate,


(4) $$\log\;r_{i}-\log\;r_{\mathrm{pa}(i)}=\phi_{i},\;\text{for}\;i\in \{1,\ldots ,2N-2\}\;\text{and}\;r_{2N-1}=1.$$

**Fig. 1.**
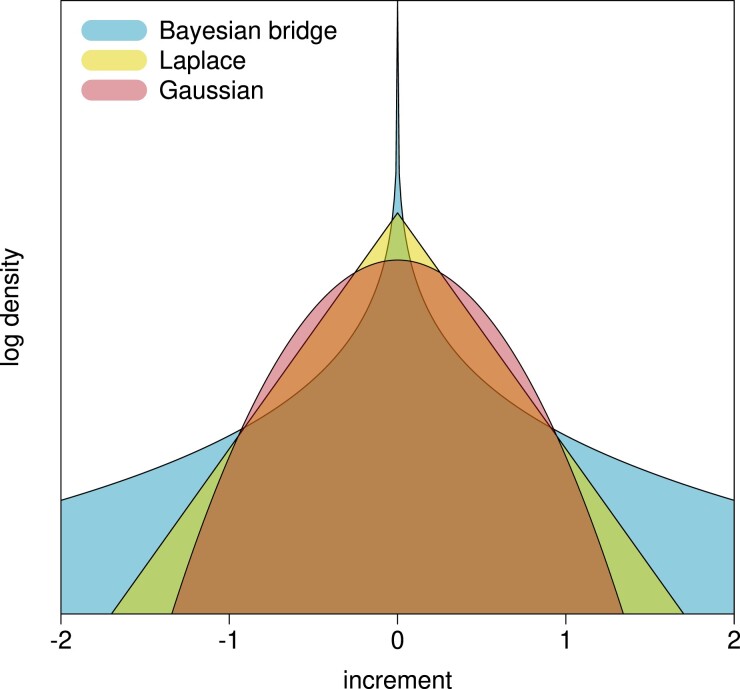
The shape of various shrinkage priors on the increment of the log-rate. The Bayesian bridge prior places more mass near 0 and has heavier tails compared to other common shrinkage priors. The Bayesian bridge reflects our a priori belief that local clocks are rare, but may arbitrarily speed up or slow down the rate of molecular evolution.

Under this parameterization, the increments $\phi =\{\phi_{1},\ldots \phi_{2N-2}\}\in R^{2N-2}$ are a linear transformation of logr. To shrink the total number of rate changes along the tree, we let $\phi_{i}\overset{\mathrm{iid}}{∼}P_{\phi }$ such that $E[\phi_{i}]=0$. Typically, $P_{\phi }$ may follow a Gaussian (Thorne et al. 1998) or Laplace distribution. We choose the flexible, heavy-tailed, Bayesian bridge prior (Polson et al. 2014) on the increments,


(5) $$P_{\phi }\propto \exp\;\left\{-{\left|\frac{\phi_{i}}{\mu }\right|}^{\alpha }\right\},$$

where μ>0 is termed the “global scale” and α∈(0,1] changes the shape of $P_{\phi }$ where smaller *α* places more mass near zero. See Fig. 1 for a comparison of the bridge to common shrinkage priors. Choosing *α* to be close to 0 forces the Bayesian bridge prior closer to best subset selection when used in a regression setting, while α=1 matches the Laplace prior. In all examples, we set $\alpha =\frac{1}{4}$ to enforce slightly stronger shrinkage than the default 0.5 employed by Polson et al. (2014). Since increments are independent, the joint prior is simply the product


(6) $$p(\phi \;|\;\mu )\propto \prod_{i=1}^{2N-2}\exp\;\left\{-{\left|\frac{\phi_{i}}{\mu }\right|}^{\alpha }\right\}.$$

### Inference

We follow the computationally efficient sampling approach outlined by Polson et al. (2014) and view the prior on the increments as a scale mixture of normals (West 1987),


(7) $$p(\phi_{i}\;|\;\mu )=\int p(\phi_{i}\;|\;\lambda_{i},\mu )d\lambda_{i},$$

where the local scale of branch *i*, $\lambda_{i}>0$ and draws from a stable distribution with positive support, see Hofert (2011), Nishimura and Suchard (2023) for more details. To improve convergence speed and maintain the benefits of our heavy-tailed prior, we employ the shrunken-shoulder regularization of Nishimura and Suchard (2023) and augment our bridge to have light tails past a reasonably large point. Our scale mixture prior on an increment becomes


(8) $$p(\phi_{i}\;|\;\lambda_{i},\mu )=\text{N}\left(0,{\left(\frac{1}{\xi^{2}}+\frac{1}{\lambda_{i}^{2}\mu^{2}}\right)}^{-1}\right),$$

where slab width *ξ* bounds the variance of increments to $\xi^{2}$. In the examples to follow, we set ξ=2, effecting a weakly informative, generous upper bound on clock rate changes. Specifically, a slab width of 2 asserts that there is at most 5% probability for $r_{i}$ to be greater than $50\times r_{\mathrm{pa}(i)}$.

We are interested in learning about the posterior,


(9) $$\begin{array}{rl} & p(r,\mu ,\gamma ,\theta ,F\;|\;S)\propto \int \underset{\mathrm{likelihood}}{\underbrace{p(S\;|\;r,\gamma ,\theta ,F)}}\\ & \underset{\mathrm{priors}}{\underbrace{p(r\;|\;\lambda ,\mu )p(\lambda )p(\mu )p(\gamma )p(\theta ,F)}}d\lambda , \end{array}$$

where $\lambda =\{\lambda_{1},\ldots ,\lambda_{2N-2}\}$, θ represents all relevant parameters that describe the molecular substitution model and, again, F is the phylogenetic tree. We place a relatively uninformative, Gamma prior on $\mu^{-\alpha }$ with shape 1 and scale 2. Additionally, we place the continuous-time Markov chain conditional reference prior of Ferreira and Suchard (2008) on the global substitution rate *γ*. This prior is uninformative and yields a proper density. We detail the priors on θ and F in each example of the sequel.

We use Markov chain Monte Carlo (MCMC) to marginalize over local scale parameters and approximate the posterior (9). Specifically, we employ a random-scan Metropolis-within-Gibbs (Levine and Casella 2006; Liu 2008) sampling approach to update the full conditional densities implicit in equation (9). Efficient sampling schemes for γ,θ,F are well described by Suchard et al. (2018), while Polson et al. (2014) outline the efficient scale-mixture approach we use to sample (μ,λ). Here, we turn our attention to sampling


(10) p(r|μ,γ,θ,F,S)∝∫p(S|r,γ,θ,F)p(r|λ,μ)dλ.

Since there are 2N−2 correlated branch-rate multipliers, one for each branch of the tree, univariable MCMC sampling schemes for r scale poorly to large trees. To remedy this difficulty, we employ HMC to sample all r simultaneously and with high acceptance probability. HMC leverages the geometry of the high-dimensional branch-rate multiplier space to propose states that are farther away than traditional proposals but stay within regions of high posterior density (HPD). HMC escapes entrapment by local extrema of the posterior by generating random momentum $\nu =\{\nu_{1},\ldots ,\nu_{2N-2}\}$ in each dimension where typically ν∼N(0,M) (Neal 2011). Often mass matrix $M=I_{2N-2}$ but HMC sampling may be improved by using an alternative M, such as an approximation of the Hessian of the log-posterior (Zhang and Sutton 2011; Stan Development Team 2017). For further reading on HMC, see Neal (2011). While HMC samplers offer more efficient posterior exploration, they require computationally expensive gradient calculations that often diminish their usefulness. Here, we exploit and extend recent work (Ji et al. 2020) on branch-specific clock rate gradients to facilitate fast inference of r under our shrinkage model.

### HMC Increment Sampler

We generate proposals in increment space, since ϕ are uncorrelated in the prior and we transform back to rate space as described by the linear transformation in equation (4). HMC sampling of the rates requires the gradient of the rate log-posterior,


(11) $$\begin{array}{rl} \frac{\partial }{\partial \phi_{k}}\log\;p(r\;|\;\mu ,\gamma ,\theta ,F,S)= & \int \frac{\partial }{\partial \phi_{k}}\underset{L[\rho (r)]}{\underbrace{\log\;p(S\;|\;r,\gamma ,\theta ,F)}}\\ & +\frac{\partial }{\partial \phi_{k}}\log\;p(r\;|\;\lambda ,\mu )d\lambda , \end{array}$$

where k∈{1,…2N−2}. To compute the gradient of the log-likelihood with respect to the increments, we first find the gradient with respect to clock rates r,


(12) $$\frac{\partial }{\partial r_{j}}L[\rho (r)]=\gamma \nabla_{\rho }L[\rho (r)]T,$$

where we compute all entries in $\nabla_{\rho }L[\rho (r)]=(\frac{\partial }{\partial \rho_{1}},\ldots ,\frac{\partial }{\partial \rho_{2N-2}})L[\rho (r)]$ with the computational O(N) algorithm derived by Ji et al. (2020) and


(13) $$T_{ij}=\left\{ \begin{array}{cc} \frac{\sum_{k}t_{k}}{\sum_{k}r_{k}t_{k}}-r_{i}t_{i}\frac{\sum_{k}t_{k}}{{\left(\sum_{k}r_{k}t_{k}\right)}^{2}} & \text{if }i=j\\ -r_{i}t_{j}\frac{\sum_{k}t_{k}}{{\left(\sum_{k}r_{k}t_{k}\right)}^{2}} & \text{if }i\neq j. \end{array}\right.$$

We complete the gradient


(14) $$\begin{array}{cc} \frac{\partial }{\partial \phi_{k}}L[\rho (r)] & =\sum_{j=1}^{2N-2}\frac{\partial }{\partial r_{j}}L[\rho (r)]\frac{dr_{j}}{d\phi_{k}}\;\\ \text{and}\;\frac{dr_{j}}{d\phi_{k}} & =\left\{ \begin{array}{cc} r_{j} & \text{if }i\text{ ancestral to }j\\ 0 & \text{otherwise}, \end{array}\right. \end{array}$$

where transformation $dr_{j}/d\phi_{k}$ follows directly from equation (4). To preserve the O(N) gradient computation, we take advantage of the tree structure explicit in equation (14) and accumulate the gradient of the log-likelihood via one postorder traversal of the tree. To begin, let *i* and *j* be both daughters of node *k* in F, then


(15) $$\;\;\;\;\;\;\frac{\partial }{\partial \phi_{k}}L[\rho (r)]=\left\{ \begin{array}{cc} r_{k}\times \frac{\partial }{\partial r_{k}}[L[\rho (r)]] & \text{if }k\text{ is a tip}\\ \left(\frac{\partial }{\partial \phi_{i}}+\frac{\partial }{\partial \phi_{j}}+r_{k}\times \frac{\partial }{\partial r_{k}}\right)L[\rho (r)] & \text{otherwise.} \end{array}\right.$$

We next turn our attention to the gradient of the log-prior,


(16) $$\frac{\partial }{\partial \phi_{k}}\log\;p(r\;|\;\lambda ,\mu )=\frac{\partial }{\partial \phi_{k}}\left[\log\;p(\phi \;|\;\lambda ,\mu )+\log\;\left|\frac{d\phi }{dr}\right|\right].$$

Since p(ϕ|λ,μ) is Gaussian, the first term gradient unwinds,


(17) $$\begin{array}{rl} \frac{\partial }{\partial \phi_{k}}\log\;p(\phi \;|\;\lambda ,\mu ) & =\frac{\partial }{\partial \phi_{k}}\sum_{j=1}^{2N-2}\log\;p(\phi_{j}\;|\;\lambda_{j},\mu )\\ & =\frac{\partial }{\partial \phi_{k}}\sum_{j=1}^{2N-2}\log\;\text{N}\left(0,{\left(\frac{1}{\xi^{2}}+\frac{1}{\lambda_{j}^{2}\mu^{2}}\right)}^{-1}\right)\\ & =-\phi_{k}\left(\frac{1}{\xi^{2}}+\frac{1}{\lambda_{j}^{2}\mu^{2}}\right). \end{array}$$

Numerical solutions to the second term in equation (11) involve the change-of-variable Jacobian $\left(\partial /\partial \phi_{k})\log\;|d\phi /dr\right|$ and appear to necessitate an $O(N^{2})$ sparse determinant computation. To facilitate faster computation of the transform, we index nodes of the tree such that i<j⟹i is not ancestral to *j*. Under this indexing, dϕ/dr is an upper triangular matrix with $1/r_{i}$ along its diagonal, see supplementary Fig. S1, Supplementary Material online for an example. The gradient of the log determinant,


(18) $$\begin{array}{rl} \frac{\partial }{\partial \phi_{k}}\log\;\left|\frac{d\phi }{dr}\right| & =\frac{\partial }{\partial \phi_{k}}\log\;\prod_{j=1}^{2N-2}\frac{1}{r_{j}}=-\frac{\partial }{\partial \phi_{k}}\sum_{j=1}^{2N-2}\log\;r_{j}\\ & =\underset{d_{k}}{\underbrace{\sum_{j}1_{\left[r_{j}\;\text{depends on }\phi_{k}\right]}}}, \end{array}$$

where 1 is the indicator function and $d_{k}$ is the number of descendants of node *k*. Altogether,


(19) $$\frac{\partial }{\partial \phi_{k}}\log\;p(r\;|\;\lambda ,\mu )=-\phi_{k}\left(\frac{1}{\xi^{2}}+\frac{1}{\lambda_{k}^{2}\mu^{2}}\right)-d_{k},$$

and we accumulate $d_{k}$ in one recursive postorder tree traversal by observing $d_{k}=d_{i}+d_{j}+1$, where, again, *i* and *j* are both daughters of *k*.

To further improve the proposals of our HMC sampler, we precondition the mass matrix M to be the current-state absolute value of the Hessian of the log-prior,


(20) $$\left|\frac{\partial^{2}}{\partial \phi_{i}\partial \phi_{j}}\log\;p(r\;|\;\lambda ,\mu )\right|=\left\{ \begin{array}{cc} \left(\frac{1}{\xi^{2}}+\frac{1}{\lambda_{i}^{2}\mu^{2}}\right) & \text{if }i=j\\ 0 & \text{otherwise.} \end{array}\right.$$

This diagonal matrix weights momentum draws by prior increment precision. Intuitively, equation (20) improves HMC sampling by rescaling increment proposals by the variance of ϕ, allowing larger steps to be taken in dimensions with larger variance. See Neal (2011) for further discussion on mass matrix transformations.

## Results

### Local Clocks in 3 Nuclear Genes of Rodents and Other Mammals

**Fig. 2.**
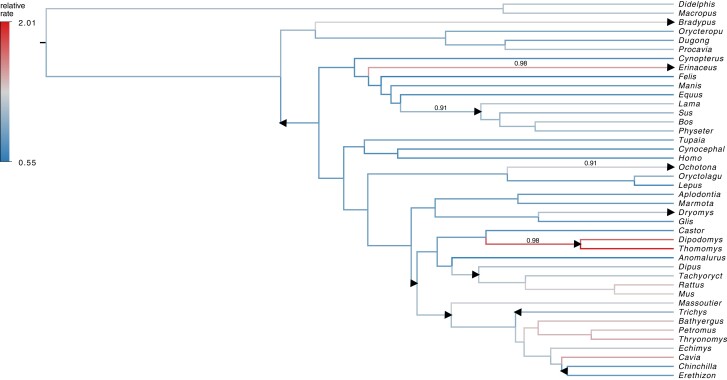
MCC tree under shrinkage clock of mammalian and rodent radiation where branches are colored by posterior mean relative clock rates r. If branch *i* starts a new clock, it is labeled with the posterior probability $\phi_{i}>0$. For comparison, local clocks of the RLC model are depicted as triangles. Triangles pointing left designate clock rate slowdown, while triangles pointing right indicate a rate increase. Two local clocks of the RLC are excluded due to topological differences between the RLC and shrinkage-clock MCC trees.

To verify the ability of our model to recover clock rate variation, we turn to a well-studied example of adaptive radiation in mammals and rodents. Huchon et al. (2002) and Douzery et al. (2003) examine the adaptive radiation of 21 rodents compared to 19 other placental mammals and two marsupial outgroups using the first two codon positions for 3 nuclear genes: ADRA2B, IRBP, and vWF (2,422 alignment sites). Douzery et al. (2003) establish the presence of clock variability within this set of taxa and report their best fitting model contains 5 “local clocks” and about 13 rate changes. Note that Douzery et al. (2003) do not require a single “local clock” to be comprised of connected components on the tree. Drummond and Suchard (2010) use the RLC model to reexamine this claim and estimate the existence of between 6 and 12 local clocks. Here, we employ our shrinkage-clock model to jointly infer the mammalian phylogeny as well as the number and location of local clocks. Because this is an ultrametric example, γ=1. We follow the specifications of Drummond and Suchard (2010) and Douzery et al. (2003) and use a general time reversible (GTR) substitution model with a 4 category discrete-*Γ* site rate model. We run 10 separate Markov chains of our shrinkage clock with 10 different starting trees for 30M states and build a maximum clade credibility (MCC) tree from the combined results (Fig. 2). We further run 100 RLC chains with 100 different starting trees for 30M states and build an MCC tree for comparison. Under the combinatorial parameter space of the RLC, we observe suboptimal mixing and that some chains converge to different modes, hence our choice for combining 100 independent chains; the 10 independent chains for the shrinkage clock simply errs on the side of caution since each independent shrinkage-clock chain converges to the same topology. Incidentally, the shrinkage-clock MCC topology differs from the RLC MCC in two places. First, *Bradypus* attaches to one of two neighbor internal branches deep in the tree. Second, *Anomalurus* is more closely related to the *Dipus* than *Castor* under the shrinkage clock. This second difference highlights the well-known difficulty in *Anomalurus* placement (Horner et al. 2007). Indeed, in an analysis of 6 nuclear genes, Blanga-Kanfi et al. (2009) group Anomaluridae closer to Dipodidae, but support for this grouping is slight and the authors remark that all 3 possible relationships among *Anomalurus*, *Dipus*, and *Castor* have been suggested in other published studies. The posterior probabilities of *Bradypus* and *Anomalurus* parent branches under the shrinkage clock are almost equal (0.64 and 0.49, respectively).

We estimate the existence of 4 local clocks (and thus about 4 rate changes) where we define a local clock on branch *i* if the posterior odds $\phi_{i}>0$ is greater than 10 or less than $\frac{1}{10}$. The posterior odds here is equivalent to a Bayes factor since an increment is equally likely to be positive or negative under the prior. While some regard a Bayes factor greater than 10 as suggestive of “strong evidence” against an alternative hypothesis (Kass and Raftery 1995), we acknowledge that this is an arbitrary cutoff and further investigation might find use for alternative cutoffs. In the approach, we do not make assumptions about the magnitude of local clocks on a tree and instead use posterior probability of increment sign to define a clock. To keep track of branch *i* across nonfixed topologies, we use the set of descendant tips of node *i* to define the branch.

To illustrate the benefits of using a heavy-tailed prior on the increments, we further compare the performance of our Bayesian bridge prior to the more usual Laplace prior for shrinkage (see Fig. 1). We again fit our shrinkage clock as described above but remove the slab and fix α=1, thus placing a Laplace prior on each increment. We find the posterior mean of increment variance under both the Laplace and Bridge priors is 0.057 with 95% HPD intervals (0.036,0.089) and (0.035,0.093), respectively. Despite having very similar variance, we find the posterior mean of the absolute maximum increment is 0.84 (0.57,1.22) and 1.01 (0.70,1.54) under the Laplace and Bridge priors, respectively. This provides evidence for induced smoothing of the clock rates under the exponential tails of a Laplace prior, that on average shrinks the largest increment by approximately 20%.

### Simulation Study

We compare the scalability and accuracy of our shrinkage clock to the RLC under a simulated example. We generate 1,000, 2,000, and 3,000 character nucleotide sequences from fixed 40, 80, and 160 tip trees 5 times each. In each simulation, there are 4 distinct lineages (A, B, C, D) of taxa, each with an equal number of tips. In all cases, time to most recent common ancestor for each lineage is 40 years and tree height is 80 years. Lineages B, C, and D evolve with a relative clock rate of 1.0, while the most recent common ancestor of lineage A starts a new clock with relative rate 2.0.

**Fig. 3.**
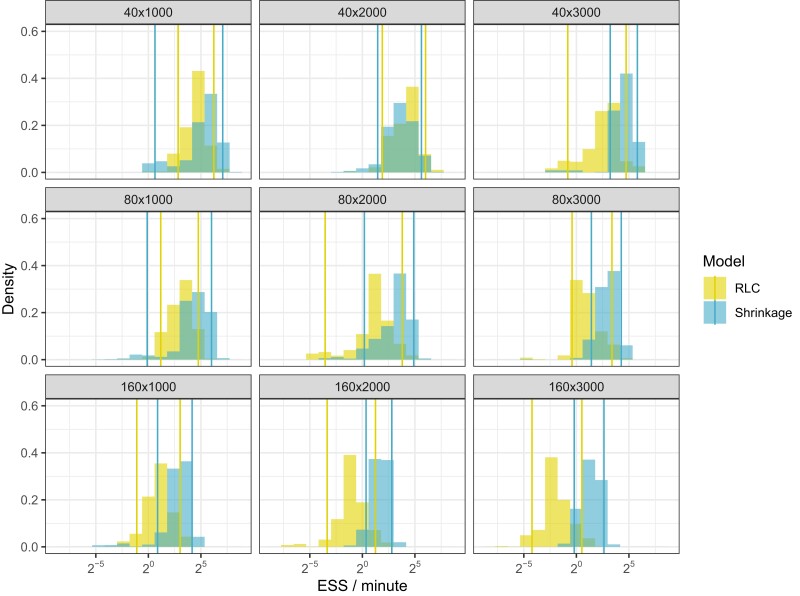
Effective sample size (ESS) of branch-specific clock rates per minute of BEAST runtime under the shrinkage clock and RLC during a full joint phylogenetic analysis. Each facet title shows data dimensions (tips × sequence length). Vertical lines show 0.05 and 0.95 quantiles for shrinkage and RLC ESS/minute.

We compare the accumulation of effective sample size (ESS) per unit time of branch-specific clock rates under both our Bayesian bridge shrinkage clock and the RLC while simultaneously inferring the phylogeny. ESS approximates the number of independent samples from a Markov chain and we use this metric to evaluate how well each inference procedure explores clock rate space. We report the results across all 45 simulated datasets in Fig. 3. We summarize median ESS/minute speed-up in Table 1 and find the shrinkage-clock speed-up scales as both the number of tips and length of sequence data increases. In all but one 40-tip example, the shrinkage clock outperforms RLC. This reveals that on certain trees with a modest number of tips, the RLC is competitive. As the tips double to 80 or quadruple to 160, the set of all possible local clocks under the RLC grows exponentially with the number of taxa (24 to 72 orders of magnitude), challenging inference under the RLC. On average, across all 45 simulations, the shrinkage clock accumulates ESS/minute 4.3× as fast.

In all examples herein, we precondition the mass matrix.

We report here that in 40 tip examples, preconditioning the mass matrix improves ESS/minute 15× for the worst-explored clock rate under the shrinkage clock.

**Table 1.** Ratio (Shrinkage:RLC) of median branch-specific clock rates ESS per minute of BEAST runtime during a full joint phylogenetic analysis

Sequence length	Number of tips		
	40	80	160
1,000	1.85	2.43	3.95
2,000	0.53	4.54	6.73
3,000	3.07	5.12	11.14

Additionally, the true relative clock rate for the “A” clade is 2.0 and we report posterior mean estimates, together with 95% HPD intervals under both our shrinkage clock and the RLC in Table 2. In all cases, the true relative clock rate is captured within the 95% HPD interval.

**Table 2.** Posterior mean and 95% HPD interval of the relative clock rate of clade “A” averaged across 5 replications for each simulation

Sequence length	# of tips	Shrinkage	RLC
1,000	40	1.71 (0.95, 2.43)	1.88 (1.00, 2.41)
2,000	40	1.71 (0.96, 2.27)	1.89 (1.40, 2.37)
3,000	40	2.15 (1.70, 2.65)	2.40 (1.22, 4.28)
1,000	80	1.89 (1.12, 2.66)	2.15 (1.56, 2.94)
2,000	80	2.05 (1.48, 2.59)	1.67 (0.99, 2.64)
3,000	80	1.98 (1.59, 2.44)	1.34 (0.99, 2.01)
1,000	160	2.05 (1.47, 2.74)	1.32 (0.98, 2.36)
2,000	160	1.96 (1.56, 2.37)	1.62 (1.00, 2.22)
3,000	160	2.04 (1.74, 2.38)	1.57 (0.99, 2.22)

The true value is 2. Here, both the shrinkage clock and RLC run for approximately equal amounts of wall time after converging to the posterior.

We further report, in Supplementary material online, posterior distributions of root age (supplementary Fig. S2, Supplementary Material online) and clade age (supplementary Fig. S3, Supplementary Material online) under each model, as well as posterior mean estimates of branch lengths (supplementary Fig. S4, Supplementary Material online). We compare these estimates to the true values we simulate from. In these simulated examples, the shrinkage clock typically exhibits higher accuracy and lower variance in parameter estimates. See the Supplementary material online for more detail.

### Influenza A Virus

We further demonstrate the scalability and utility of our shrinkage-clock model by examining the evolution of two major influenza A virus (IVA) surface glycoprotein subtypes: hemagglutinin (HA) H7, and neuraminidase (NA) N7. Both HA and NA protein mutations impact IVA’s epitope and allow IVA to escape adaptive immune responses (Wilson and Cox 1990; McAuley et al. 2019). Worobey et al. (2014) find divergence time estimation is sensitive to molecular clock model specification. To consistently estimate divergence times, Worobey et al. (2014) allow the clock rates of various glycoprotein subtypes to vary only between viral hosts and find H7 and N7 each evolve slower in equine hosts than avian hosts. We reexamine this claim with our more general shrinkage-clock model that does not assume the existence of host-dependent clock rates. Specifically, we re-analyze 146 complete gene (1,716 nt) sequences of H7 and 92 complete gene (1,416 nt) sequences of N7. In each case, we follow the model specifications of Worobey et al. (2014) and employ a GTR substitution model with 4 category discrete-*Γ* site rate model. We depart from their example, however, in our use of tree prior. We employ a Bayesian skygrid prior (Gill et al. 2013) with 50 population size bins and a cutoff of 200 years instead of using the skyride prior (Minin et al. 2008).

**Fig. 4.**
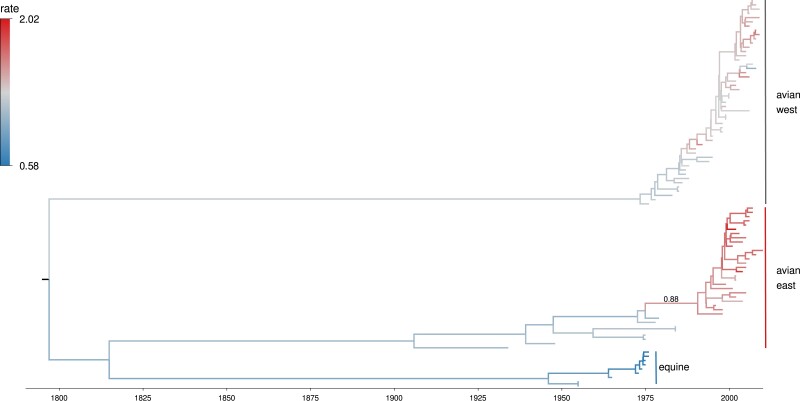
MCC tree for influenza A’s NA subtype N7. Branches are colored by posterior clock rates. The most probable local clock is reported and labeled with posterior probability that $\phi_{i}>0$. The second most probable clock starts a subclade of the equine lineage and has a Bayes factor $\phi_{i}>0$ of 0.351.

**Fig. 5.**
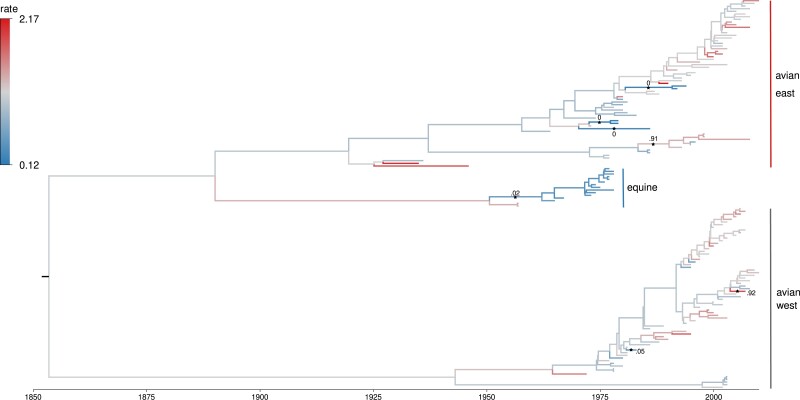
MCC tree for influenza A’s HA subtype H7. Branches are colored by posterior clock rates. Local clocks are labeled with a star and the posterior probability $\phi_{i}>0$.

We find no sharp local clocks exist with Bayes factor >10 or $<\frac{1}{10}$ on the NA N7 tree under our shrinkage-clock model but do see evidence for rate heterogeneity. Incidentally, the most likely clock occurs on the branch that begins the Eastern avian clade of the NA N7 tree (Fig. 4). The second most probable clock is found in a subclade of the equine lineage. On the other hand, we estimate the existence of 7 local clocks on the HA H7 tree and report these in Fig. 5. Overall, the mean posterior clock rate for NA N7 is lower than HA H7. We report the posterior mean and 95% HPD intervals of *γ* are $2.7\;\{2.1-3.3\}\times 10^{-3}$ and $3.3\;\{2.8-3.9\}\times 10^{-3}$ for NA N7 and HA H7, respectively. Additionally, under our shrinkage clock, the posterior mean root dates and 95% HPD intervals of the N7 and H7 trees in absolute time are 1798 (1733–1855) and 1853 (1808–1897), respectively. Furthermore, the median ESS/minute is .50 and .11 for the N7 and H7 examples respectively. While the size of this example is comparable to our simulation study, additional modeling considerations slow the speed of inference. In this example, the RLC fails to converge to the posterior when run for an equivalent amount of time.

## Discussion

We develop a robust autocorrelated heritable clock rate model that scales to large trees, avoids excessively shrinking clock rates and learns the number and location of local clocks on a tree. Crucially, we model the incremental difference between log clock rates on the tree as drawing from a Bayesian bridge prior that shrinks most changes to approximately 0 unless the data warrant otherwise. To facilitate scalability, we employ HMC to generate proposals in the independent increment space and derive recursive postorder algorithms to compute the gradient and its requisite transforms. Our recursive algorithms achieve O(N) computational speed, signifying that they will continue to work well as *N* grows large. We further improve the speed of our HMC sampler by preconditioning the mass matrix with the Hessian of the log-prior.

In our examination of the adaptive radiation of rodents and other mammals (see the section “Local Clocks in 3 Nuclear Genes of Rodents and Other Mammals”), our shrinkage clock recovers the location of 4 local clocks estimated under the RLC. Douzery et al. (2003) also recover these 4 rate changes in addition to several more. We note that our shrinkage clock prefers branches with similar clock rates to be connected. However, we do not classify some rate heterogeneity as new local clocks due to our particular choice of classification threshold. We choose a Bayes factor of 10 to classify clocks, but shrinkage-clock users may wish to adjust this threshold to increase or decrease clock rate sensitivity. Comparing the statistical properties of different clock-classification schemes remains an important avenue for future work.

We apply our shrinkage-clock model to reexamine the number of local clocks present in IVA surface glycoproteins NA N7 and HA H7 across equine and avian hosts. We confirm the equine slowdown reported by Worobey et al. (2014) but interestingly find that the N7 tree shows marked rate variation (Fig. 4) between western and eastern hemisphere avian influenza lineages, however, this rate variation is not supported by the Bayes factor cutoff. Root date estimates vary from Worobey et al. (2014) but this may be in part due to the different tree priors. Additionally, we find 7 local clocks under our shrinkage clock across the H7 tree. Since 3 of these clocks belong to edges of tip nodes, this may reflect incomplete sampling or sequencing error. Despite inferring 6 more clocks than the host-specific model, the posterior mean estimate of root date under our shrinkage clock is within 5 years of previous estimates (Worobey et al. 2014).

Our shrinkage clock accumulates ESS/minute of clock rates 4.3× faster on average than the RLC across a variety of simulated examples. If ESS is used as a stopping criterion for phylogenetic reconstruction, we expect this may save shrinkage clock users 76% of BEAST runtime on average compared to the RLC. As the bridge exponent *α* approaches 0, the bridge prior density is more peaked near zero resulting in sharper increment shrinkage and thus better distinguishable local clocks. Nishimura and Suchard (2023) examine multiple *α* and report closer to optimal coverage for smaller *α* but with increasing computational cost due to mixing. For this reason, shrinkage-clock users may find it useful to adjust *α* depending on desired clock rate coverage or to tackle even larger tree studies.

## Supplementary Material

msad242_Supplementary_Data

## Data Availability

We provide installation instructions for the development branch of BEAST in which we implement our model and make all BEAST XML files used in this work publicly available at https://github.com/suchard-group/shrinking_clocks.
