# Supplementary material for: Shrinkage-based Random Local Clocks with Scalable Inference
Source: Mol Biol Evol. 2023 Nov 10;40(11):msad242. doi: 10.1093/molbev/msad242 (PMC10665039; doi:10.1093/molbev/msad242)
Supplement: msad242_Supplementary_Data [file msad242_supplementary_data.pdf]

Shrinkage-based random local clocks with scalable inference

Supplementary material

Fast gradient illustration

In Figure (S1) below, we illustrate our indexing scheme that leads to fast gradient computation.

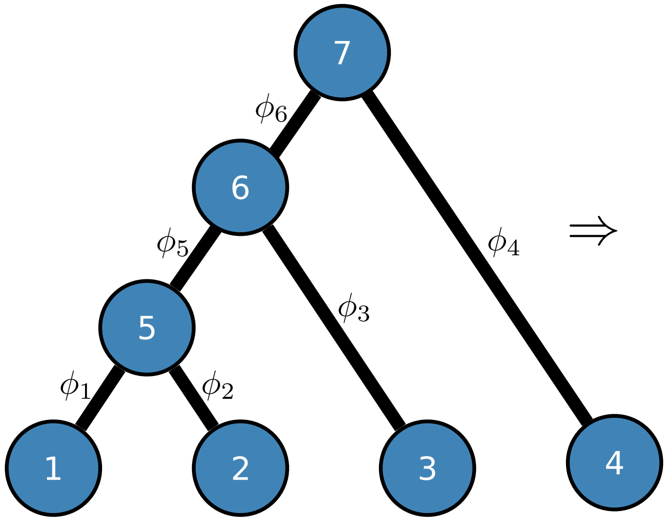

$$\Longrightarrow \quad \frac{d\boldsymbol{\phi}}{d\mathbf{r}} = \begin{bmatrix} \frac{1}{r_1} & 0 & 0 & 0 & -\frac{1}{r_5} & 0 \\ 0 & \frac{1}{r_2} & 0 & 0 & -\frac{1}{r_5} & 0 \\ 0 & 0 & \frac{1}{r_3} & 0 & 0 & -\frac{1}{r_6} \\ 0 & 0 & 0 & \frac{1}{r_4} & 0 & 0 \\ 0 & 0 & 0 & 0 & \frac{1}{r_5} & -\frac{1}{r_6} \\ 0 & 0 & 0 & 0 & 0 & \frac{1}{r_6} \end{bmatrix}$$

Figure S1: Example tree with corresponding Jacobian matrix. Index $i < j \implies i$ is not ancestral to j thus the Jacobian is upper-triangular and the determinant is the product of diagonal entries.

Simulation results

One of the principal goals of local clock models is to better estimate divergence times, or the ages of specific nodes in the tree. Below we report the accuracy of important node dates as well as branch time estimates under our simulation study. Specifically, we compare estimates under both the shrinkage-clock and random local clock (RLC) to the actual simulated values. At the top of each subplot we define the number of tips N, times the length of molecular sequence data M, in NxM format. For each NxM combination, we simulate five separate

data sets on the same fixed tree. In Figures (S2, S3), we remove burn-in from both shrinkage and RLC chains and plot results across all five replicates while controlling for runtime.

In Figure (S4), we run each chain for 7.5 M steps and compute the maximum clade credibility (MCC) tree after removing burn-in. We subsequently plot each posterior mean branch time from the MCC tree against its corresponding branch time in the true tree from which we simulate the data. We match corresponding branches by identifying branches according to the set of tips descendant from a given branch. Each point we plot is a posterior mean of a specific branch under a given replicate and we again visualize all five replicates.

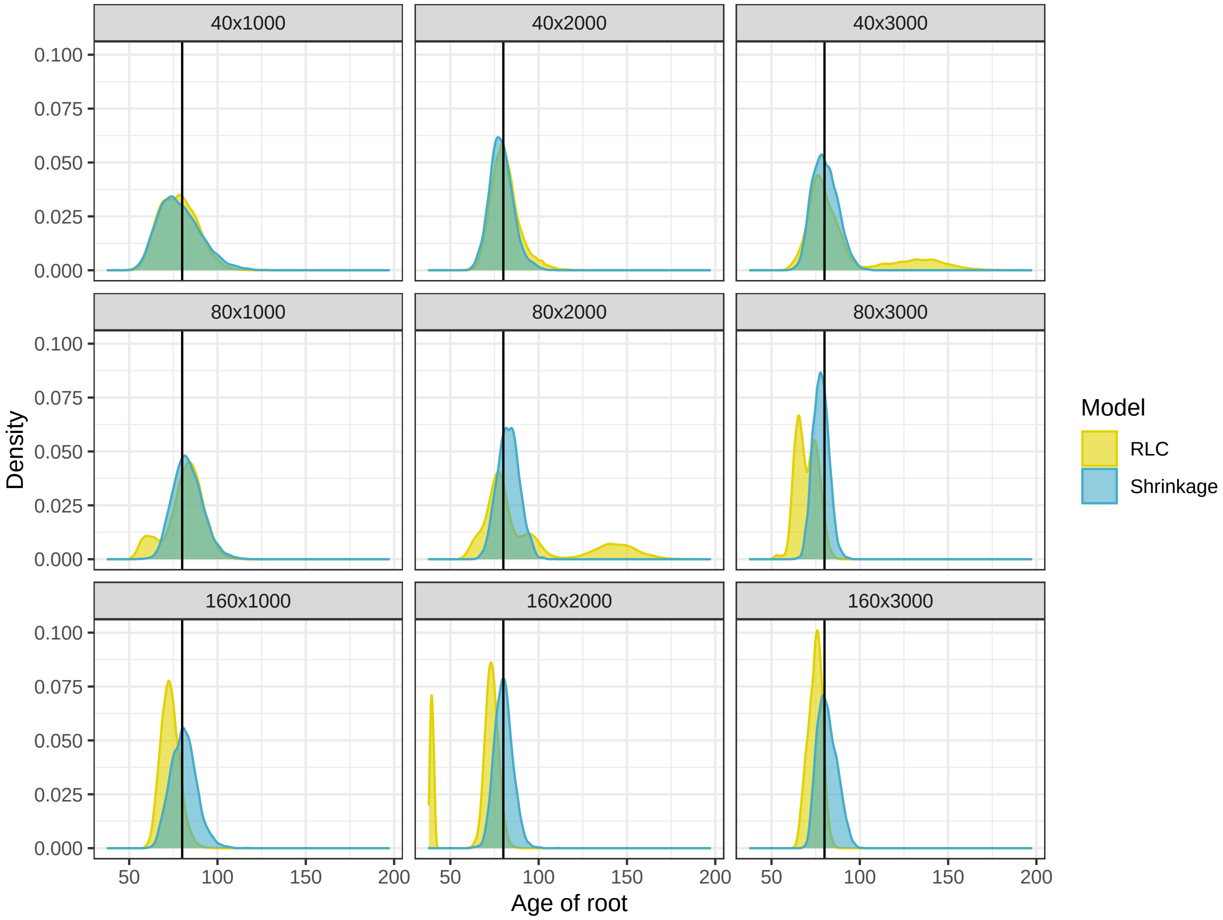

Figure S2: Posterior distribution of the age of the root in arbitrary units of time. True age (80) is represented by the vertical black lines.

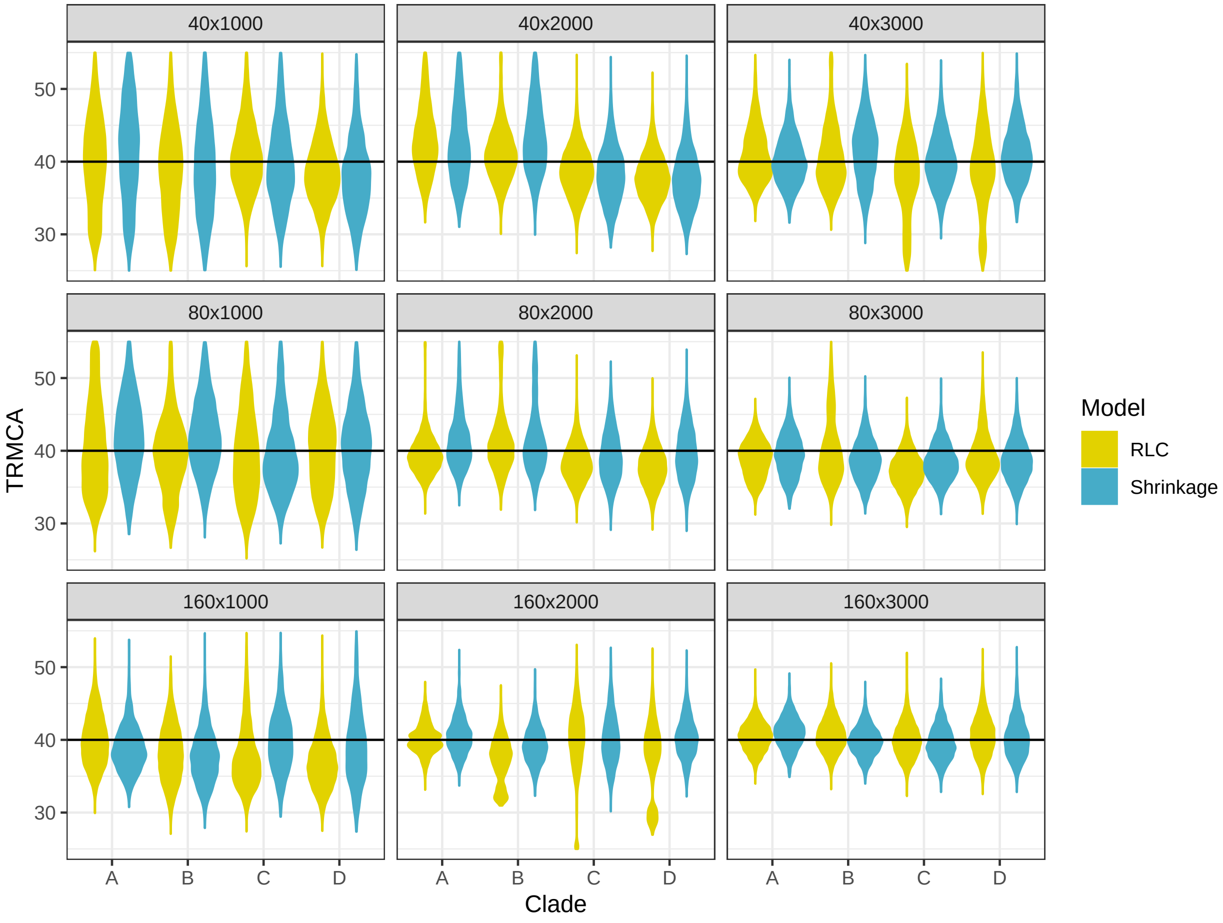

Figure S3: Posterior distributions of time to most recent common ancestor (TMRCA) of clades A, B, C and D. The true TMRCA of each clade (40) is represented by the horizontal black lines.

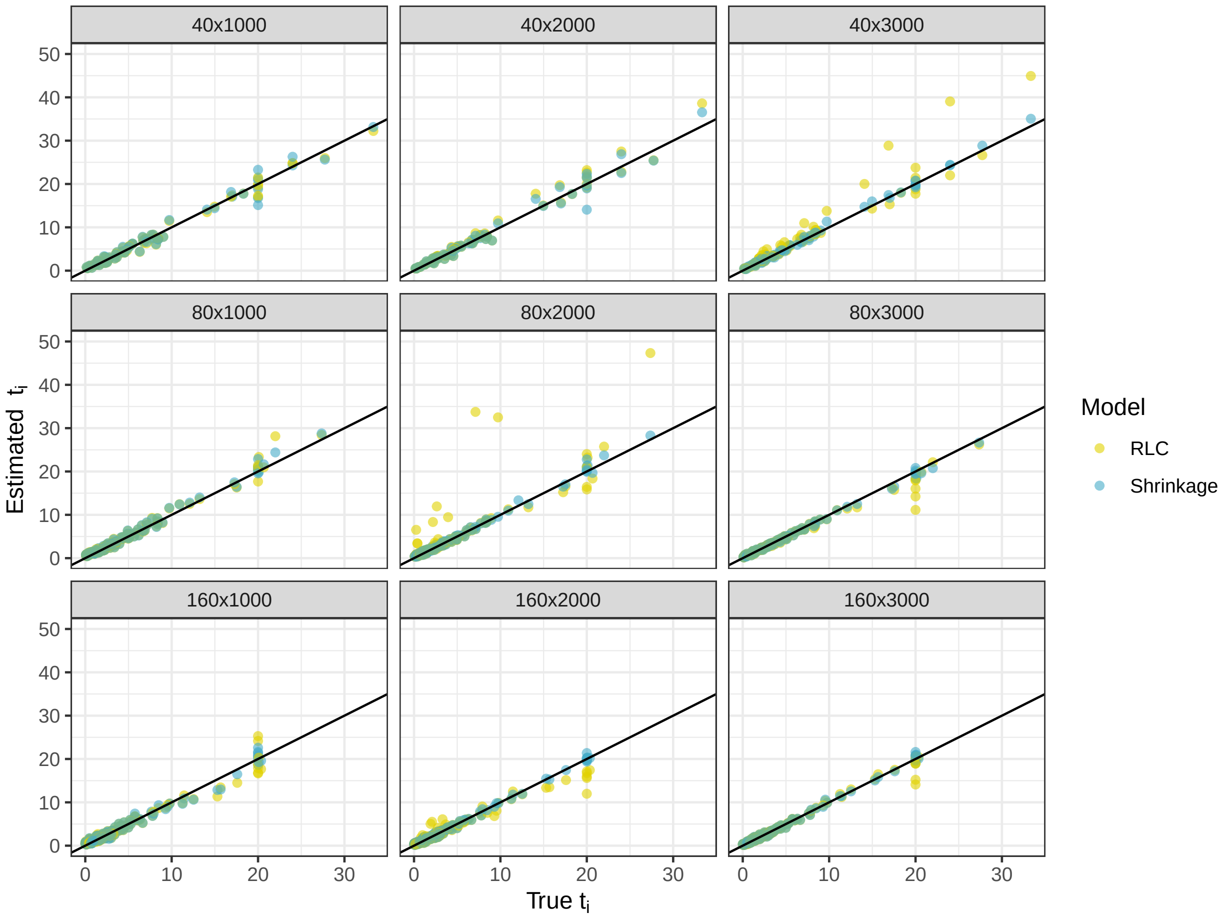

Figure S4: Posterior mean branch time vs true branch time, computed across all five replicates. Each branch is uniquely identified by its set of descendant tips. The black line shows the idealized fit where estimated branch time is precisely equal to the true branch time.

Multi loci simulation

In this section we demonstrate that our model may be applied to massive data sets with multiple loci and provide a reference XML in our GitHub repository to re-create this analysis. We consider a single 40 tip tree that is again broken into 4 clades (A, B, C, D), with 10 tips each. The MRCA of the A clade starts a local clock with relative rate that is twice the clock rate of the B, C and D clades. We assume this local clock structure applies uniformly to each loci, but one may alternatively choose to learn about multiple local clocks in parallel.

Table 1: Posterior means and 95% HPD intervals of average relative clock rates on a clade. The true relative clock rate for the A clade is 2 while the the true clock rate for clades B, C and D are identically 1.

Clade	Avg. clock rate
A	1.98 (1.90, 2.16)
B	0.99 (0.95, 1.02)
C	1.01 (0.97, 1.05)
D	1.00 (0.97, 1.04)

For each tip, we simulate a 100 thousand nucleotide (nt) sequence from 100 different HKY substitution models, each one generating 1000 nt. As in the simulation study above, to choose parameters to simulate each substitution process, we draw from the BEAUti default priors. Specifically, we generate the HKY transition-transversion rates κ from a lognormal distribution with mean 1 and standard deviation 0.25. We generate the frequencies of the HKY from a Dirichlet distribution characterized by parameter $\alpha = [1, 1, 1, 1]$.

We fit our shrinkage clock and report ESS/minute of the branch-specific clock rates in Figure S5 to capture the speed of inference under our model. As expected, the ESS/minute is on the same scale as the single locus 40 tip examples with smaller sequence alignments. Indeed, we expect ESS/minute to be somewhat lower since a full joint phylogenetic inference under this multi loci example takes at least 2.3$\times$ longer (19.8 minutes vs 8.5 minutes per million states) to generate the same number of samples from the posterior as the single locus, 1000 nt counterpart from the previous simulation study. Furthermore, we provide posterior estimates of the average relative clock rate for each clade in Table (1). Notice in this example, that HPD intervals are tighter than in the previous simulated examples and still cover the true parameter value.

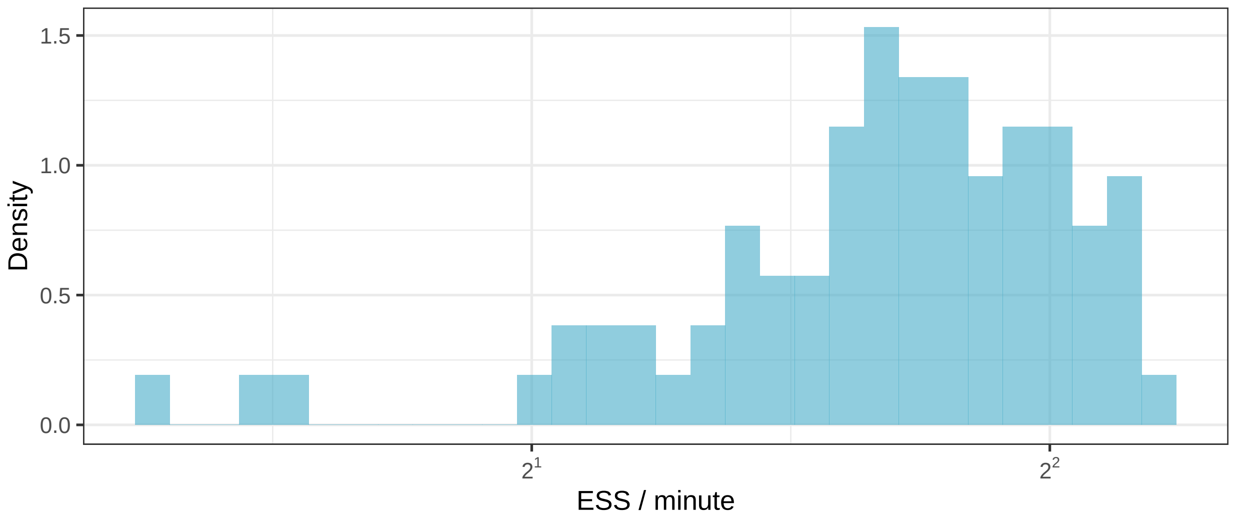

Figure S5: Effective sample size (ESS) of branch-specific clock rates per minute of BEAST runtime under the shrinkage clock during a full joint phylogenetic analysis of a 40 tip tree with a sequence alignment containing 100 loci, each locus with 1000 nt.

Implementation

Here we validate our implementation of the shrinkage clock in BEAST by computing, in two separate ways, the two new components of our model: our Bayesian bridge prior density and the gradient of the posterior. To verify our implementation we consider a 40 tip example from our simulation study.

We evaluate the log joint prior density of the branch rates using 100 posterior samples of the branch rates, and local- and global-scale parameters. Since the Bayesian bridge shrinkage prior is a scale mixture of normals, we compare the log joint density evaluation in BEAST to evaluation using dnorm() in R. We find the average absolute relative difference between the two to be 2.02×10^{-16}. Separately, we compare our analytic gradient to a numerical central-difference gradient computed in BEAST and find that the maximum absolute relative difference between gradient vectors is 1.92×10^{-5}.
